# OXGR1-Dependent (Pro)Renin Receptor Upregulation in Collecting Ducts of the Clipped Kidney Contributes to Na^+^ Balance in Goldblatt Hypertensive Mice

**DOI:** 10.3390/ijms251810045

**Published:** 2024-09-18

**Authors:** Pilar Cárdenas, Camila Nuñez-Allimant, Katherin Silva, Catalina Cid-Salinas, Allison C. León, Zoe Vallotton, Ramón A. Lorca, Lilian Caroline Gonçalves de Oliveira, Dulce E Casarini, Carlos Céspedes, Minolfa C. Prieto, Alexis A. Gonzalez

**Affiliations:** 1 Institute of Chemistry, Pontificia Universidad Catoólica de Valparaióso, Valparaióso 2340000, Chile; pilar.cardenas@pucv.cl (P.C.); camila.nunez.a01@mail.pucv.cl (C.N.-A.); katherin.silva.q@mail.pucv.cl (K.S.); catalina.cid.s@mail.pucv.cl (C.C.-S.); allison.leon.l@mail.pucv.cl (A.C.L.); 2 Department of Physiology, Tulane Hypertension and Renal Center of Excellence, Tulane University School of Medicine, New Orleans, LA 70112, USA; zvallotton@tulane.edu (Z.V.); mprieto@tulane.edu (M.C.P.); 3 Division of Reproductive Sciences, Department of Obstetrics and Gynecology, University of Colorado Anschutz Medical Campus, Aurora, CO 80045, USA; ramon.lorca@cuanschutz.edu; 4 Departamento de Medicina, Disciplina de Nefrologia, Escola Paulista de Medicina, Universidade Federal de São Paulo, São Paulo 04023-062, Brazil; lilian.oliveira@unifesp.br (L.C.G.d.O.); casarini.elena@unifesp.br (D.E.C.); 5 Faculty of Medicine and Science, Universidad San Sebastián, Santiago 7510602, Chile; carlos.cespedes@uss.cl

**Keywords:** tricarboxylic cyclic acid pathway, kidney, renal blood flow, prorenin, 2K1C

## Abstract

The two-kidney, one-clip (2K1C) Goldblatt rodent model elicits a reduction in renal blood flow (RBF) in the clipped kidney (CK). The reduced RBF and oxygen bio-ability causes the accumulation of the tricarboxylic cycle intermediary, α-ketoglutarate, which activates the oxoglutarate receptor-1 (OXGR1). In the kidney, OXGR1 is abundantly expressed in intercalated cells (ICs) of the collecting duct (CD), thus contributing to sodium transport and electrolyte balance. The (pro)renin receptor (PRR), a member of the renin–angiotensin system (RAS), is a key regulator of sodium reabsorption and blood pressure (BP) that is expressed in ICs. The PRR is upregulated in 2K1C rats. Here, we tested the hypothesis that chronic reduction in RBF in the CK leads to OXGR1-dependent PRR upregulation in the CD and alters sodium balance and BP in 2K1C mice. To determine the role of OXGR1 in regulating the PRR in the CDs during renovascular hypertension, we performed 2K1C Goldblatt surgery (clip = 0.13 mm internal gap, 14 days) in two groups of male mice: (1) mice treated with Montelukast (OXGR1 antagonist; 5 mg/Kg/day); (2) OXGR1^−/−^ knockout mice. Wild-type and sham-operated mice were used as controls. After 14 days, 2K1C mice showed increased systolic BP (SBP) (108 ± 11 vs. control 82 ± 5 mmHg, *p* < 0.01) and a lower natriuretic response after the saline challenge test. The CK group showed upregulation of erythropoietin, augmented α-ketoglutarate, and increased PRR expression in the renal medulla. The CK of OXGR1 knockout mice and mice subjected to the OXGR1 antagonist elicited impaired PRR upregulation, attenuated SBP, and better natriuretic responses. In 2K1C mice, the effect of reduced RBF on the OXGR1-dependent PRR upregulation in the CK may contribute to the anti-natriuretic and increased SBP responses.

## 1. Introduction

Growing evidence demonstrates that renin, prorenin, and the (pro)renin receptor (PRR) play important roles in the activation of the intrarenal renin–angiotensin system (RAS) [1,2,3]. The PRR is a member of the RAS that binds prorenin and renin to form angiotensin (Ang) I [4] and, due to the abundant expression of ACE along the nephron [5], intratubular Ang II as well. In the collecting duct, the PRR regulates sodium transport via the epithelial sodium channel (ENaC) [6,7] as well as Ang II type 1 receptors (AT1R)-dependent stimulation of ENaC and renin in the distal nephron segments [8].

Augmented synthesis of the PRR in distal nephron segments, particularly in the collecting duct, has been described in experimental animal models of hypertension including chronic Ang II infusions [9,10,11,12,13], renin transgenic rats [14], and 2K1C Goldblatt hypertensive rat models [15,16]. PRR upregulation is also associated with augmented intratubular prorenin and renin levels in the same experimental hypertensive models [16,17].

While we know that the mechanisms of activation of the systemic RAS are mediated by renin secretion from juxtaglomerular cells when RBF is reduced in the clipped kidney in 2K1C Goldblatt hypertensive models, the role of intermediaries of the tricarboxylic acid cycle in RAS regulation is unknown. Furthermore, the long-term effects of chronic reductions in renal blood flow have mostly been described in rats [17,18,19]. Reduced blood flow and oxygen levels cause the suppression of the tricarboxylic acid cycle and α-ketoglutarate is channeled toward gluconeogenesis. The accumulation of Nicotinamide Adenine Dinucleotide (NADH) decreases the activity of α-Ketoglutarate dehydrogenase, leading to α-Ketoglutarate accumulation and release [18,19]. During low oxygen availability in cardiac and neural tissues, the reaction α-Ketoglutarate to glutamate occurs in a reverse manner (glutamate to α-Ketoglutarate), leading to α-Ketoglutarate accumulation [20,21].

α-Ketoglutarate binds to a receptor that was initially described as an orphan receptor known as GPR99; it was then deorphanized due to its ability to bind α-Ketoglutarate and is now called oxoglutarate receptor 1 (OXGR1) [22]. Northern blot analysis with human tissues revealed that GPR99 mRNA is expressed in the kidney and placenta [23]. OXGR1 is abundantly expressed in type B and non-A–non-B intercalated cells [24]. Concentrations of α-Ketoglutarate ranges in physiological concentrations in the urine [25]. Moreover, in vitro studies showed that α-Ketoglutarate stimulates pendrin activity and electroneutral reabsorption of NaCl in the CD cells [25]. OXGR1 actions can be specifically inhibited by the leukotriene receptor antagonist Montelukast [26]. We have demonstrated that α-Ketoglutarate treatment in inner medullary collecting duct (IMCD) cells can increase intracellular Ca^2+^ and upregulate PRR expression; these effects can be prevented by a pharmacological blockade of OXGR1 [27]. There is no evidence about the role of OXGR1 on PRR regulation during reduced blood supply to the kidney and its impact on the physiological regulation of sodium excretion and intrarenal RAS regulation.

The present study was designed to determine the role of OXGR1 in the expression of the PRR, sodium handling, BP, and intrarenal Ang II in the 2K1C Goldblatt mouse model for 14 days, assessing the effect of pharmacological blockade of OXGR1 (Montelukast) and by using knockout mice with specific deletion of OXGR1. We tested the hypothesis that chronic reduction in renal blood flow contributes to the accumulation of α-Ketoglutarate, OXGR1-dependent upregulation of PRR, activation of intratubular RAS, augmentation of intrarenal Ang II, and stimulation of sodium reabsorption, which increases blood pressure. 

## 2. Results

### 2.1. 2K1C Goldblatt Model Using a 0.13 mm Internal Gap in Mice Evidenced Chronic Reductions in Renal Blood Flow Even at 14 Days

**Figure 1:**
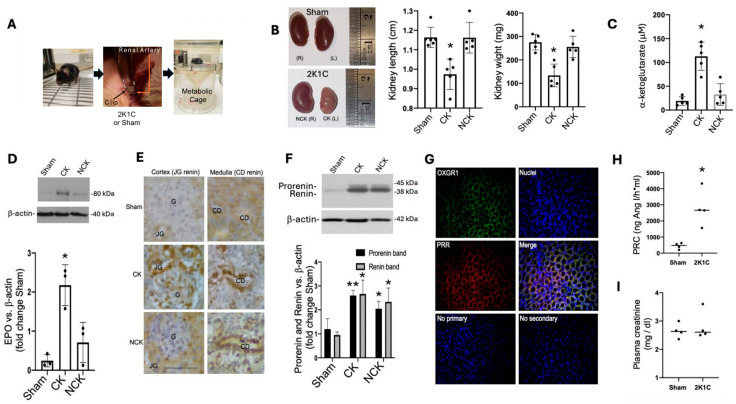
Control sham-operated and mice with 2K1C surgery were housed in metabolic cages for urine collection. A silver clip with an internal diameter of 0.13 mm was placed around the left artery for 14 days (**A**). After 14 days of chronic reduction in blood flow, clipped kidneys showed reduced size and weight as compared to sham mice (**B**). Intrarenal levels of α-Ketoglutarate in the clipped kidney were significantly augmented as compared to control (sham) kidneys (**C**). Reduced blood supply for 14 days was also evidenced by the increased abundance of erythropoietin (EPO) protein in the clipped kidney as compared to sham-operated mice, * *p* < 0.05; versus sham group (**D**). Chronic reduction in renal blood flow caused the upregulation of juxtaglomerular (JG) renin, even at day 14 in the clipped kidney (CK). In contrast, renin was not augmented in the non-clipped kidney (NCK). Collecting duct (CD) renin was augmented in both clipped and non-clipped kidneys (**E**). As previously demonstrated by our group, medullary prorenin and renin protein bands can be detected in medullary tissues. Immunoblotting showed that prorenin and renin bands are upregulated in the clipped and non-clipped kidneys after 14 days (**F**). Co-localization of OXGR1 and PRR in medullary collecting ducts was confirmed by immunofluorescence using specific antibodies—OXGR1 (Alexa Fluor 488, Green color) and PRR (Alexa Fluor 594, red color), leading to merge-yellow color (**G**), scale bar 50 µm. Specific controls with omission of the first or secondary antibody and nuclei staining with DAPI demonstrated the specificity of the experiment. In previous protocols using whole trunk blood collection, we showed that plasma renin content (PRC) is augmented in 2K1C mice, as compared to sham mice (**H**). No changes were observed in blood creatinine. * *p* < 0.05; ** *p* < 0.01 versus sham group, n = 5 for (**B**,**C**,**F**). For (**H**,**I**) n = 4, *p* < 0.05 versus sham group, scale bar 50 µm. For F and H * *p* < 0.05 versus sham group.

As shown in Figure 1A, the animals were housed in metabolic cages for urine collection. For 2K1C surgery, a silver clip with an internal diameter of 0.13 mm was placed around the left artery. After recovery, mice were subjected to a protocol consisting of 14 days of chronic blood reduction and a saline challenge on day 10; measurements during the study included water intake, urine excretion, and food intake on the day before the euthanasia and saline challenge. The left clipped kidney displayed a reduced size and weight compared to kidneys from sham mice (Figure 1B). Intrarenal levels of α-Ketoglutarate in the clipped kidney were significantly augmented as compared to the control (clipped: 112.8 vs. sham: 19.2 μM *p* < 0.05) and no differences were found between non-clipped and kidneys from sham mice (non-clipped: 23.1 vs. sham: 19.2 μM, *p* = non-significant), as seen in Figure 1C. Since renal erythropoietin synthesis increases in response to decreases in renal perfusion, we further tested the abundance of erythropoietin protein in the clipped kidney (Figure 1D). Erythropoietin was augmented in the clipped kidney but not in the non-clipped kidney. We also examined the abundance of renin labeling in Juxtaglomerular cells on day 14. Renin immunostaining was increased in juxtaglomerular cells and substantially decreased in the non-clipped kidneys (Figure 1E), which is an indicator of systemic RAS. We found that prorenin and renin (and prorenin) immunostaining was increased in both the clipped and non-clipped medullary tissues (Figure 1E, right, middle, and lower panels). As shown previously, prorenin and renin bands can be detected in medullary tissues and mostly correspond to collecting duct source [28,29]. Furthermore, increases in collecting duct renin in 2K1C rats have previously been reported [16,17,30]. Prorenin and renin bands were augmented in medullary tissues of clipped and non-clipped kidneys of 2K1C mice (Figure 1F). Importantly, these tissues are free from the contribution of glomeruli and juxtaglomerular renin. By day 14, systolic blood pressure was increased in 2K1C mice compared to the sham group (108 ± 11 vs. control 82 ± 5 mmHg, *p* < 0.01). Co-localization of OXGR1 and the PRR in ICs was assessed by immunofluorescence using specific antibodies. OXGR1 (Alexa Fluor 488, Green color) and the PRR (Alexa Fluor 594, red color) co-localized in renal collecting ducts (Figure 1G). Specific controls with omission of the first or secondary antibody and nuclei staining with DAPI demonstrated the specificity of the experiment. In accordance with Figure 1E, plasma renin content was augmented in 2K1C mice (Figure 1H). No changes in plasma creatinine were found in the 2K1C group (Figure 1I).

### 2.2. OXGR1 Antagonist Montelukast Attenuated the Increases in Blood Pressure

**Figure 2:**
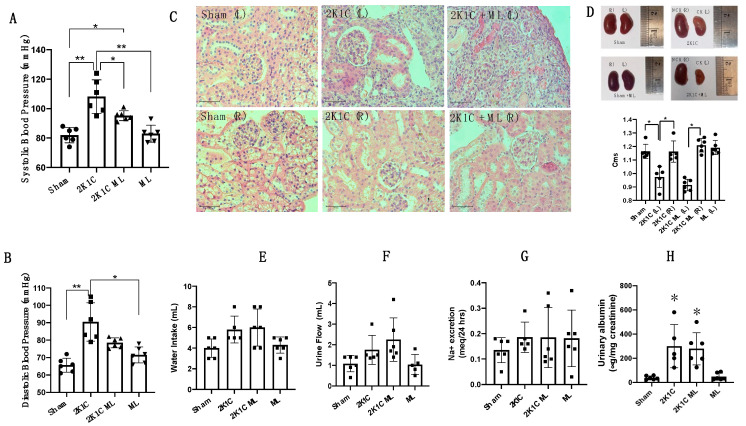
OXGR1 antagonist Montelukast (ML) attenuated the increases in systolic blood pressure in 2K1C (**A**), and blunted the increases in diastolic blood pressure in 2K1C mice (**B**). ML mice were not different from controls. Clipped kidneys (CKs) and kidneys from 2K1C + ML mice showed an enlarged Bowman’s capsule; 2K1C + ML mice showed hyaline substances (**C**). 2K1C surgery led to a reduction in the size of the CKs; 2K1C + ML mice showed no observable size difference compared to 2K1C alone (**D**). Goldblatt surgery did not change water intake (**E**), urine flow (**F**), or 24 h sodium excretion (**G**) after 14 days. To verify kidney injury signs shown in (**C**), we performed albumin measurements in urine. Data show that, after 2 weeks of 2K1C surgery, there was an increased urinary albumin/creatinine ratio in the 2K1C and 2K1C + ML groups as compared to the sham surgery group (* *p* < 0.05), while ML treatment did not have any effect (**H**). ** *p* < 0.01 versus sham group in B. scale bar 50 μm.

The 2K1C Goldblatt mice displayed increased systolic blood pressure. Therefore, we further examined the blood pressure response of chronic OXGR1 blockade using Montelukast delivered by subcutaneous osmotic minipumps at a rate of 5 mg/Kg/day in 2K1C mice. Blood pressure was evaluated between days 12 and 13 of treatment. As shown in Figure 2A,B, the increase in systolic blood pressure was partially attenuated in 2K1C mice administered Montelukast (2K1C + ML) (95 ± 3 vs. control 82 ± 5 mmHg, *p* < 0.05), as well as 2K1C mice not treated with ML. ML-treated mice did not differ from controls (83 ± 6 vs. control 82 ± 5 mmHg, *p* = NS). Similar results were found in diastolic blood pressure. The 2K1C mice showed increased diastolic blood pressure compared to controls (91 ± 11 vs. control 65 ± 4 mmHg, *p* < 0.05), but no difference was found in the 2K1C + ML or ML-treated mice compared to controls.

### 2.3. Altered Histology and Decreased Kidney Size Caused by Clip Implantation after 14 Days Were Not Altered by OXGR1 Antagonism

Compared to sham-operated kidneys, clipped kidneys from 2K1C mice showed enlarged glomeruli (2K1C: 53 ± 5 vs. control: 45 ± 4 μm, *p* < 0.05) and Bowman’s spaces (2K1C: 12 ± 4 vs. control: 8 ± 2 μm, *p* < 0.05). In left kidneys from the 2K1C + ML group, we observed similar results (2K1C + ML: 11 ± 5 μm, *p* < 0.05 vs. controls). We also observed less preserved structure and the presence of hyaline substances in the clipped kidneys of mice subjected to RBF reduction (Figure 2C). Glomerular size and Bowman’s space size were also augmented in the right kidneys from 2K1C mice (2K1C: 49 ± 3 vs. controls: 41 ± 3 μm, *p* < 0.05) and 2K1C + ML mice (2K1C + ML: 53± 5 μm, *p* < 0.05 vs. control). Changes in the structural characteristics were related to changes in kidney size, as shown in Figure 2D. 2K1C surgery caused a reduction in kidney size; no changes were observed in 2K1C + ML mice as compared to 2K1C alone. Kidney weight was reduced in 2K1C mice treated with ML to the same extent than that observed in the 2K1C group, and no statistical differences were found (2K1C: 133 ± 21 mg vs. 2K1C + ML: 139 ± 35 mg, *p* = non-significant).

### 2.4. 2K1C Goldblatt Surgery Does Not Change Water Intake, Urine Flow, or 24 h Sodium Excretion after 14 Days

To test whether 2K1C Goldblatt surgery and co-treatment with Montelukast affect water intake, urine flow, or 24 h sodium excretion, the mice were maintained in metabolic cages for 24 h before the day of the euthanasia, and urine was collected. As shown in Figure 2E–G, none of the evaluated parameters were changed among the groups.

### 2.5. Upregulation of PRR in the Clipped Kidney Is Blunted by OXGR1 Antagonism

**Figure 3:**
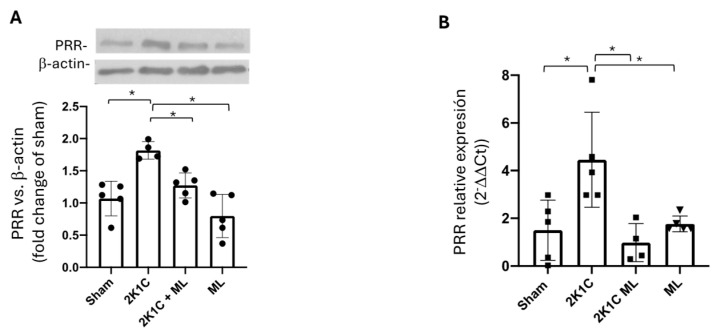
Upregulation of PRR and intrarenal Ang II in the clipped kidney was blunted by OXGR1 antagonist Montelukast (ML). PRR protein levels (**A**) and mRNA abundance (**B**) were increased in the clipped kidney, while ML treatment for 14 days prevented this effect. * *p* < 0.05 versus sham group.

The PRR is upregulated in rats with renovascular hypertension [15] and plays a significant role in the development and progression of hypertension [12,31,32]. We evaluated the effects of the reduced renal blood flow and OXGR1 blockade on protein and mRNA levels of PRR in the clipped kidney. Protein levels of the PRR were augmented in the clipped kidney; however, chronic pharmacological blockade of OXGR1 was able to prevent this increase (Figure 3A). Similar results were observed when the expression of mRNA levels of the PRR were analyzed according to each group (Figure 3B). The PRR was augmented in the non-clipped kidneys in 2K1C and 2K1C + ML mice. No changes were observed in the ML mice (Figure S1). No significant differences were found in mRNA levels in non-clipped kidneys (Figure S1). 

### 2.6. Intrarenal Ang II Levels Were Increased in the Clipped Kidney While OXGR1 Antagonisms Prevented This Effect

It has been shown that ACE activity is altered in the 2K1C Goldblatt model [16], which may lead to changes in intrarenal Ang II levels. We tested the effect of OXGR1 pharmacological blockade on intra-renal Ang II. Table 1 shows that intrarenal ACE activity was increased in the clipped and non-clipped kidneys from 2K1C mice. The pharmacological antagonism of OXGR1 was not able to prevent this effect in the right kidney; however, a slight but not significant increase was observed in the clipped kidney. Intrarenal Ang II was greatly augmented in non-clipped kidneys of the 2K1C mice, while the pharmacological blockade of OXGR1 partially impaired this effect. In the left kidneys of 2K1C mice, Ang II levels were also increased; OXGR1 blunted this effect. No effects were observed in mice treated with Montelukast alone. 

### 2.7. Pharmacological Blockade of the OXGR1-Enhanced Natriuresis in Mice with 2K1C Surgery

**Figure 4:**
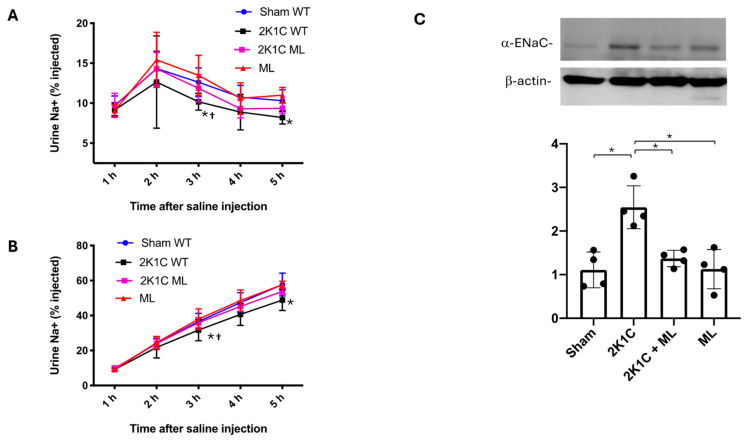
Mice were subjected to a saline load challenge to test the ability of the mice to excrete a saline load on day 10. The sodium excretion was significantly lower in 2K1C mice at 3 and 5 h post-injection as compared with wild-type (sham WT) mice (**A**). Similar results were observed when analyzed by cumulated sodium excretion (**B**). Co-treatment with Montelukast (ML) was able to prevent this effect. Since ML prevented PRR upregulation and augmentation of intrarenal Ang II levels, the expression of ENaC might likely be altered. 2K1C surgery caused an increased ENaC expression in the clipped kidney that was blunted by ML (**C**). * *p* < 0.05 2K1C versus sham; ^†^ *p* < 0.05 versus 2K1C + ML mice.

Reduced blood perfusion in the clipped kidney activates systemic RAS and upregulation of the intrarenal enzymatic machinery to form intratubular Ang II, promoting sodium reabsorption [16,17,30]. To evaluate the role of OXGR1 on sodium handling, we tested the effect of OXGR1 pharmacological blockade in sham and 2K1C animals subjected to a sodium challenge. Close to the end of the protocol (day 10), we evaluated the natriuretic response to an extracellular volume expansion by using a saline challenge (see Section 4). At the 3 and 5 h time points, the sodium excretion was significantly lower in the 2K1C group compared with the control (sham) wild-type mice (*p* < 0.05, Figure 4A). Co-treatment with Montelukast was able to prevent this effect at 3 and 5 h time points, showing significant differences with 2K1C mice (*p* < 0.05, see Figure 4A). This was also shown by analyzing cumulated sodium excretion at 5 h (Figure 4B).

### 2.8. Enhanced Natriuretic Responses to Saline Challenge Are Associated with ENaC Expression

Reduced blood flow in the clipped kidney causes OXGR1-dependent PRR upregulation and intrarenal Ang II formation. This might be responsible for impaired natriuretic responses and the upregulation of distal sodium channels. We evaluated the expression of ENaC in the clipped kidney. As shown in Figure 4C, αENaC was significantly augmented in the clipped kidney of 2K1C mice, while the chronic treatment with Montelukast prevented this effect. The αENaC subunit expression was augmented in the non-clipped kidneys of 2K1C mice and 2K1C + ML mice (Figure S2).

### 2.9. Mice with Whole Genetic Ablation of OXGR1 (Oxgr1^−/−^) Did Not Show Increases in Blood Pressure after Goldblatt 2K1C Surgery

**Figure 5:**
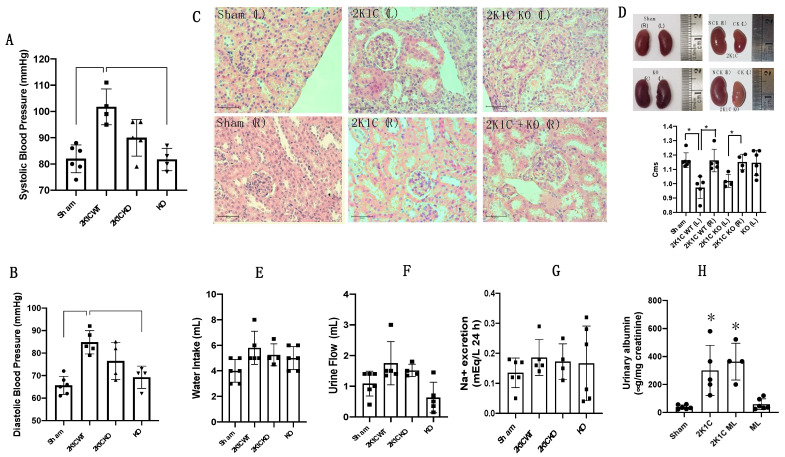
Increases in systolic (**A**) and diastolic (**B**) blood pressure were prevented in OXGR1 knockout (KO) mice subjected to 2K1C surgery. KO mice did not differ from controls regarding diastolic or systolic blood pressure. The enlarged distance from Bowman’s capsule distance to glomeruli was observed in the left clipped kidney (L) of 2K1C and 2K1C KO mice, and the same comparative image from 2K1C in Figure 2 is shown (**C**). The reduction in clipped-kidney size was also observed in KO mice subjected to the 2K1C surgery and did not differ from wild-type mice (**D**). No changes were observed in water intake (**E**), urine flow (**F**), and 24 h sodium excretion (**G**) among the groups after 14 days. Kidney injury signs showed in C were confirmed by the results showing increased urinary albumin/creatinine ratio in 2K1C and 2K1C KO mice as compared to sham surgery group (* *p* < 0.05). No changes were observed in KO mice (**H**). Scale bar 50 μm.

We analyzed the effect of the systemic genetic ablation of the Oxgr1 gene on systolic blood pressure responses after 13 days of Goldblatt 2K1C surgery. As shown in Figure 5A,B, the increased systolic and diastolic blood pressure in the 2K1C group was not observed in *Oxgr1*^−/−^ knockout mice (systolic: 90 ± 8 vs. control 82 ± 5 mmHg, *p* = NS; diastolic: 76 ± 8 mmHg vs. control 65 ± 4 mmHg, *p* = NS). No difference was found in the control (sham) group and KO sham-operated mice (systolic: 81 ± 4 vs. 82 ± 5 mmHg, *p* = NS; diastolic: 69 ± 5 vs. 65 ± 4 mmHg, *p* = NS). This result indicates that the absence of OXGR1 function impacts blood pressure. Of note, we present data from four animals, since two animals died at the end of the protocol and were not considered for biochemical and molecular analysis.

### 2.10. Altered Histology and Decreased Kidney Size Caused by Clip Implantation after 14 Days Was Not Altered in Oxgr1^−/−^ Mice

Increased glomeruli size, Bowman space size, and tubular dilation observed in clipped and non-clipped kidneys from mice subjected to Goldblatt surgery were also observed in *Oxgr1*^−/−^ knockout mice at the same intensity (2K1C KO: 51 ± 6 vs. control: 45 ± 4 μm, *p* < 0.05; Bowman capsule distance to glomeruli (2K1C KO: 13 ± 6 vs. control: 8 ± 2 μm, *p* < 0.05). Similarly, hyaline substances were also observed in clipped kidneys of *Oxgr1^−/−^* knockout mice subjected to a reduction in renal flow (Figure 5C). The reduction in clipped-kidney size was also observed in KO mice subjected to the 2K1C surgery and did not differ from wild-type 2K1C mice (Figure 5D). Similarly, kidney weight was reduced in 2K1C KO mice to the same extent as that observed in 2K1C mice (2K1C: 133 ± 21 g vs. 2K1C KO: 129 ± 41 g, *p* = non-significant).

### 2.11. 2K1C Goldblatt Surgery Does Not Change Water Intake, Urine Flow, or 24 h Sodium Excretion after 14 Days in Oxgr1^−/−^ KO Mice

As observed in the WT and WT 2K1C Goldblatt mice treated with the pharmacological blockade of the OXGR1, no differences were found between WT controls (sham) mice and floxed (Oxgr1^+/−^) mice subjected or not to Goldblatt surgery with regard to water intake, urine flow, and 24 h sodium excretion. As observed in the WT and WT 2K1C Goldblatt mice treated with the pharmacological blockade of the OXGR1, no differences were found between wild-type control (sham) mice and floxed (*Oxgr1*^+/−^) mice subjected or not to Goldblatt surgery with regard to water intake, urine flow, and 24 h sodium excretion, as shown in Figure 5E–G. To confirm the evidence of kidney damage, we measured the albumin/creatinine ratio in urine. The data demonstrated that, 2 weeks after 2K1C, there was an increase in the urinary albumin/creatinine ratio, which was observed to the same extent as in the KO mice (Figure 5H).

### 2.12. Upregulation of PRR in the Clipped Kidney Was Blunted in Oxgr1^−/−^ KO Mice

**Figure 6:**
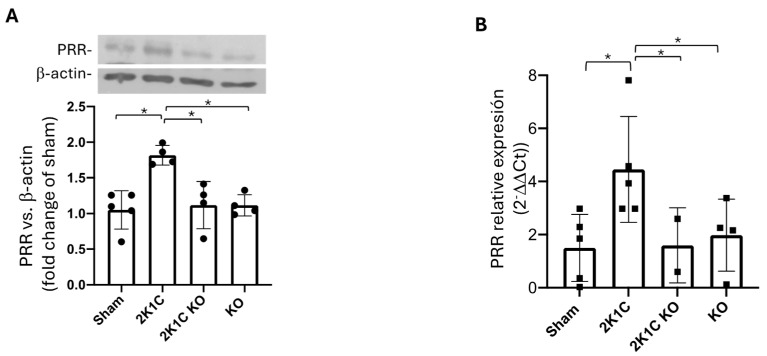
Upregulation of PRR in the clipped kidneys of 2K1C mice was not evidenced in OXGR1 knockout (KO) mice. PRR protein levels (**A**) and mRNA abundance (**B**) were increased in the clipped kidneys but not in OXGR1 KO mice. * indicates statistical differences (*p* < 0.05 between the groups compered).

The PRR was augmented in 2K1C mice (the same 2K1C group previously analyzed); however, the PRR protein levels in the clipped kidney from *Oxgr1^−/−^* mice were not statistically different from those of the controls (Figure 6A). Similarly, no differences were found in the mRNA levels (Figure 6B). In the non-clipped kidneys, the PRR was similarly augmented in the 2K1C and 2K1C KO groups (Figure S1). Also, no differences were found in floxed *Oxgr1^+/−^* mice compared to wild-type mice.

### 2.13. Intrarenal Ang II and ACE Activity in Wild-Type and Oxgr1^−/−^ KO 2K1C Mice

We tested the effect of the absence of OXGR1 on intra-renal Ang II and ACE activity. Table 1 shows that ACE activity is augmented in the clipped (left) and non-clipped (right) kidneys, while in Oxgr1^−/−^ 2K1C mice this effect is suppressed. ACE activity was slightly higher than for the controls in the non-clipped kidneys; however, it was not significant. Renal Ang II was augmented in the non-clipped and clipped kidneys, while Oxgr1^−/−^ 2K1C mice did not show differences in the clipped kidney (left) as compared to controls. Right kidneys from Oxgr1^−/−^ 2K1C mice showed slightly higher Ang II levels than the controls. No effects were observed in the group treated with Montelukast alone. 

### 2.14. Oxgr1^−/−^ Evidenced a More Rapid Natriuretic Response Compared with 2K1C Mice after Sodium Challenge Which Is Also Associated with ENaC Expression

**Figure 7:**
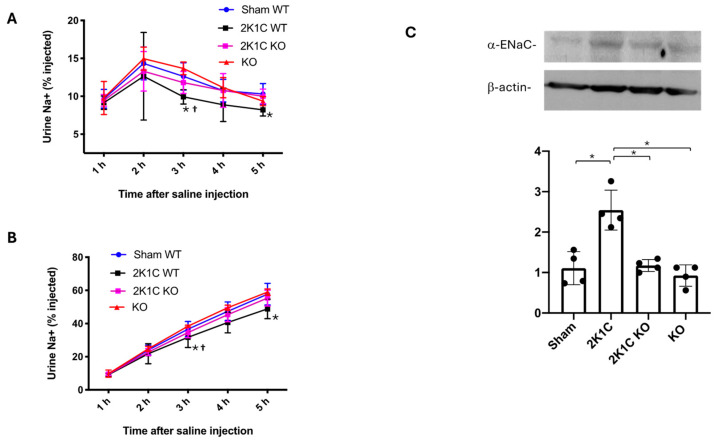
The ability to excrete a saline load was evaluated in wild-type (WT) mice with sham or 2K1C surgery and in OXGR1 knockout (KO) mice. Urine was collected for 5 h in metabolic cages. Sodium excretion was expressed as the amount of the sodium load excreted during the 5 h observation period. The reduced sodium excretion evidenced in the 2K1C group at 3 and 5 h was not observed in 2K1C KO mice (**A**); the same results were observed for accumulated sodium excretion (**B**). As described before, 2K1C surgery caused an increased ENaC expression in the clipped kidneys that was not observed in KO mice (**C**). * *p* < 0.05 2K1C versus sham; ^†^ *p* < 0.05 versus 2K1C + ML group.

Mice lacking OXGR1 displayed similar natriuretic responses at 3 and 5 h time points compared to wild-type mice. However, Oxgr1^−/−^ mice subjected to 2K1C surgery excreted a greater amount of sodium (percentage of injected) and a more rapid natriuretic response compared to 2K1C wild-type mice (Figure 7A). The results of cumulated urine sodium excretion as a percentage of injected also showed the same trend, with significant differences between 2K1C and sham WT mice and between 2K1C KO versus 2K1C groups (Figure 7B). Reduced blood flow in the clipped kidney causes OXGR1-dependent PRR upregulation and intrarenal Ang II formation, which, again, might be related to changes in ENaC abundance. As shown in Figure 7C, the alpha subunit of ENaC was significantly augmented in the clipped kidney of wild-type 2K1C mice; however, *Oxgr1*^−/−^ mice did not display changes in αENaC abundance compared to wild-type sham mice.

## 3. Discussion

In the present study, using the Goldblatt two-kidney one-clip mouse model, we demonstrated the following: (1) reduced renal blood flow increases α-ketoglutarate levels in the clipped but not in the non-clipped kidneys; (2) PRR protein and mRNA levels are augmented in the clipped kidney, but blockade of the α-ketoglutarate receptor, OXGR1, by using the pharmacological antagonist montelukast, can prevent the PRR upregulation; (3) clipped kidneys from *Oxgr1*^−/−^ mice do not show changes in PRR levels; (4) increased blood pressure, impairment of natriuretic responses, and increased intrarenal Ang II and αENaC protein levels were attenuated in mice with pharmacological OXGR1 antagonism and in *Oxgr1*^−/−^ mice. 

We validated the 2K1C experimental mouse model by assessing and verifying the characteristics of reduced clipped kidney size (Figure 1B), increases in blood pressure 14 days post-Goldblatt surgery, and effect of reduction in renal blood on the stimulation of erythropoietin expression in the clipped kidney (Figure 1D). The 2K1C rodent model is characterized by the continuous activation of systemic RAS due to the stimulation of juxtaglomerular renin in the clipped kidney, as observed in Figure 1F. Reduced renal blood flow-dependent systemic RAS activation also stimulates intratubular RAS, collecting duct renin (Figure 1F), plasma renin content (Figure 1H), Ang II formation, and ENaC-dependent sodium reabsorption, which finally impacts blood pressure. Unilateral renal artery clipping for 14 days causes increases in systolic blood pressure and plasma renin activity in rats and ACE activity [33,34]. While juxtaglomerular renin is upregulated in the clipped kidney and downregulated in the non-clipped kidney, in the experimental model of reno-vascular hypertension, renin in the collecting duct is upregulated in both non-clipped and clipped kidneys [16]. The PRR plays a relevant role in the activation of the intrarenal and intratubular RAS [8]. During chronic Ang II infusion, transgenic mice with ablation of PRR in the collecting duct display blunted increased systolic blood pressure, decreased sodium reabsorption, decreased levels of Ang II, and active renin in the urine [31,35]. Mice with nephron-specific deletion of PRR exhibit urine concentration defect [32]; this phenomenon may reflect the specific role of the PRR on intrarenal sodium and water transport. 

In the collecting duct, the PRR is regulated by Ang II through Ca^2+^ and PKC pathways [36]. Due to the fact that OXGR1 is a Gq-coupled GPCR [37], Ca^2+^ and PKC are likely involved in the signaling pathways in intercalated cells to control tubular sodium and water transport. We recently reported that α-ketoglutarate promotes the upregulation of the PRR in primary cultures of inner medullary collecting duct (IMCD) cells [27]. The upregulation of the PRR in IMCD cells during high glucose conditions [38] is partially mediated by the activation of OXGR1 [27], which is supported by previous observations showing that the accumulation of NADH decreases the activity of α-ketoglutarate dehydrogenase, leading to α-ketoglutarate accumulation and release [19] along with the accumulation of other intermediaries of the Krebs cycle [39]. Tokonami et al. described the OXGR1 signaling and actions in the collecting duct and their impact on sodium transport [25]. OXGR1 is expressed in type B and non-A–non-B intercalated cells, co-localizing with the PRR (Figure 1G). The α-ketoglutarate can be detected at physiological concentrations in the urine able to activate the OXGR1 [25]. Micro-perfusion experiments showed that α-ketoglutarate added to isolated cortical collecting ducts promoted pendrin-dependent electroneutral reabsorption of NaCl. These mechanisms may be relevant in conditions of reduced blood supply to the kidney. A reduction in blood flow to the kidney results in decreased oxygen levels, leading to metabolic alterations [40]. In this condition, oxidative phosphorylation in the mitochondria slows down due to the reduced availability of oxygen, which serves as the final electron acceptor in this process, causing the reactions of this chain to occur more slowly. In complex II or succinate dehydrogenase, a lower level of succinate is oxidized to fumarate, leading to a succinate accumulation [18,41]. The buildup of succinate slows down the Krebs cycle, consequently leading to an accumulation of ⍺-ketoglutarate. This metabolite is a substrate of 2-oxoglutarate-dependent dioxygenases (2OGDDs). These enzymes are pivotal in mediating the adaptation to hypoxia through the hypoxia-inducible factor pathway [42]. 

**Figure 8:**
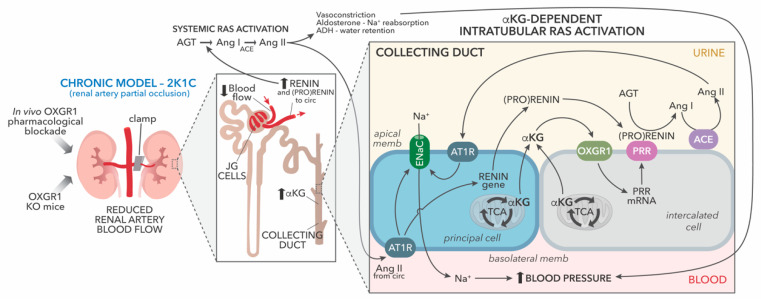
Proposed hypothetical model showing the effect of chronic partial obstruction of the left renal artery by using the 2-kidney, 1-clip, 2K1C model. Increased synthesis and release of renin from juxtaglomerular (JG) cells promotes systemic renin–angiotensin system (RAS) activation and enhance distal tubular sodium (Na^+^) reabsorption through basolateral/blood Ang II type 1 receptor (AT1R) via epithelial Na^+^ channel (ENaC). Chronic reductions in oxygen bio-ability were evidenced by increased levels of erythropoietin at 14 days. Systemic RAS activation also promotes upregulation of prorenin and renin in principal cells of the collecting duct via AT1R and binding of prorenin or renin to the PRR in the neighbor intercalated cell. Reduced blood flow leads to the accumulation of α-ketoglutarate. We propose that, during reduced blood flow, α-ketoglutarate activates the collecting duct OXGR1 receptor, leading to the upregulation of PRR enhancing the activity of renin and prorenin, stimulating intra-tubular Ang II formation and activation of AT1R promoting Na^+^ reabsorption.

The renal medulla is mostly composed of medullary collecting ducts, which have a special environment characterized by high osmolality and hypoxia. Anaerobic glycolysis in the deeper medullary collecting ducts leads to the accumulation of Krebs cycle metabolites such as succinate and ⍺-ketoglutarate, promoting the activation of succinate receptors (SUCNR1) and OXGR1, and promoting the activation of intracellular signaling pathways related to hypoxia [39,43]. Hypoxic conditions caused by the reduction in renal blood flow may be able to activate these intracellular pathways that influence sodium transport impacting blood pressure. Several pathways may contribute to the regulation of intrarenal RAS in these conditions. Renin synthesis and release is stimulated by internal baroreceptors in renin-secreting cells of the juxtaglomerular apparatus due to a reduction in the afferent arteriole pressure associated with a decrease in the renal blood flow. Renin release facilitates the conversion of Ang I from angiotensinogen (AGT), which is further metabolized to Ang II. Ang II then reaches the renal tubules to stimulate renin synthesis through basolateral AT1 receptors. Another condition includes reduced blood flow, causing the accumulation of ⍺-ketoglutarate and the activation of OXGR1 to increase the PRR in the apical membrane of the intercalated collecting duct cell. Since AGT is also formed in the proximal tubule and filtered from the plasma, it serves as a substrate for renin which might be present in the collecting duct fluid to form Ang I. Due to the presence of ACE along the nephron, intratubular Ang II will likely be boosted by both mechanisms (Figure 8). Although some of the intracellular pathways related to OXGR1 in distal nephron segments have been described, it will be important to identify the mechanisms associated with metabolic signaling that interact with the intratubular RAS and the influence of other hormones such as aldosterone, endothelin, prostaglandins, bradykinin, among other molecules, with important functions in the collecting duct. Currently, our group is investigating whether OXGR1 and the PRR are integrated central pathways for water and sodium transport in principal and intercalated cells.

Although the soluble PRR (sPRR) is also augmented and detected during experimental models of hypertension [10,44,45,46] and correlates with renal function in essential hypertensives [47], other studies suggested that serum sPRR concentration is independent of blood pressure levels and of renin and prorenin concentrations in patients with very diverse degrees of RAS activity and also in patients with diabetes [48]. Other studies demonstrated an association between sPRR levels in humans and mice with heart failure characterized by water–sodium retention and reduced when water–sodium retention is suppressed [49]; furthermore, it has been proposed that serum levels of sPRR are also related to chronic kidney disease [50]. Although most of the studies are focused on the association and correlations between sPRR levels in plasma, we focused our studies on the full-length PRR and its essential role in urine concentration, as we previously reported [31]. Undoubtably, the role of the sPRR needs to be considered for complementary further studies. 

Our data show that PRR and ENaC expression were markedly elevated in 2K1C mice, whereas in 2K1C KO mice, PRRT and ENaC protein levels were suppressed to the same level as observed in wild-type sham mice or control KO mice. In contrast, increases in systolic and diastolic blood pressure were not fully suppressed, indicating that other mechanisms may be responsible for maintaining the elevated blood pressure. Of note, if OXGR1^−/−^ mice is a whole knockout model, then the systemic influence of the absence of the OXGR1 gene may also be responsible for these results. Although we do not demonstrate the systemic RAS parameters in all groups, previous evidence in controls and 2K1C mice showed that Goldblatt surgery increased plasma renin content, which was in line with the increased renin immunolabeling in juxtaglomerular cells. One of the limitations of this study was the absence of blood data regarding Ang II, aldosterone, sPRR, plasma prorenin, α-ketoglutarate, and other vasoactive molecules, which may be the focus of future research. 

In recent reports, it has been suggested that the ability of sodium–glucose transporter (SGLT2) inhibitors in the proximal tubules relies on the increased delivery of α-ketoglutarate to the distal segments [51] and particularly to the collecting ducts wherein the OXGR1 is abundantly expressed [25]. The decreased glucose entry into the proximal tubule cell may cause increased gluconeogenesis and bioavailability to secrete α-ketoglutarate, which is also linked to the synthesis of ammonia impacting on the acid base. OXGR1 stimulates electroneutral sodium reabsorption in coordination with the activity of Cl-/HCO3- exchange through pendrin [25]. However, it is also demonstrated that the direct addition of α-ketoglutarate to cortical-collecting duct cells exerts an inhibitory effect on ENaC [25]; however, it is not clear what are the mechanisms of regulation during the reductions in renal blood flow and oxygen supply to the clipped kidney nor the effects of these intricated mechanisms of regulation in the non-clipped kidney, which is subjected to high pressure and high blood levels of Ang II [52,53]. It seems that the summary of these effects causes salt reabsorption, which is attenuated by the pharmacological blockades of the OXGR1 or by the absence of the OXGR1 receptor, as shown in the experiments using the *Oxgr1^−/−^* mice. 

Most of the data collected from the 2K1C rats suggest that, although one kidney should be enough to maintain fluid and sodium balance and normal arterial pressures, the non-clipped kidney develops an impaired renal autoregulatory capability and enhanced tubular sodium reabsorption [54,55]. Moreover, despite renin downregulation during the first days following clipping, the clipped and non-clipped kidneys have augmented intrarenal Ang II and ACE activity, and this effect is observed even in the long term (several weeks) [16,56]. Increases in circulating Ang II concentrations during the first 1–2 weeks of 2K1C hypertension cause systemic effects; however, it has been shown that, after several weeks of stenosis, renal perfusion pressure to the clipped kidney can be re-established, and plasma renin activity and circulating Ang II concentrations tend to return to the normal levels even though the arterial pressure remains elevated. Here, we demonstrated that plasma renin content is augmented after 2 weeks of 2K1C surgery (Figure 1H), which is in accordance with previous results [56] and agrees with the observed increases in blood pressure. Previous data using whole blood trunk collection also demonstrated no changes in plasma creatinine (Figure 1I). However, histological structural changes in clipped and non-clipped kidneys were associated with increased albuminuria, an effect that has previously been observed in a similar protocol by using a 0.12 internal gap [57]. This also suggests that the activation of RAS may contribute to kidney damage. Then, during this maintenance phase, Ang II continues to exert powerful actions on the function of the clipped and non-clipped kidneys. Interestingly, we observed that, even after 14 days of stenosis evidenced by kidney size reduction, juxtaglomerular renin was still higher than in the control and non-clipped kidneys. Erythropoietin protein expression was also elevated, suggesting that the renal perfusion pressure to the clipped kidney was not re-established at 14 days using the 0.13 mm clip. Despite this, the data confirm that our 2K1C mouse model for 14 days displays increased blood pressure, reduced blood flow, and activation of intrarenal RAS. Intriguingly, intrarenal ACE activity and tissue Ang II content were not augmented in 2K1C OXGR 1^−/−^ mice (Table 1), which suggests that other mechanisms may be involved in intrarenal RAS activity during reduced blood pressure. 

In conclusion, our study demonstrates that, in 2K1C mice, the effect of reduced renal blood flow in the clipped kidney is enough to increase intrarenal ⍺-ketoglutarate levels, causing the OXGR1-dependent PRR upregulation associated with the increased alpha ENaC protein abundance. The fact that OXGR1 is abundant in the kidney and that ORXG1 might determine PRR-dependent Ang II formation/Na^+^ reabsorption opens new important information that can be used to develop new drugs, therapeutic (novel OXGR1 inhibitors), and diagnostic approaches for the treatment of hypertension, cardiovascular, and kidney disease.

## 4. Materials and Methods

### 4.1. Animals

Mice of C57BL/6J background were used for the studies in accordance with the Declaration of Helsinki and bioethical protocol described, approved by the Institutional Review Board from the Pontificia Universidad Católica de Valparaíso (protocol code BIOEPUCV-BA 482-2022). Mice were divided according to the specific protocol using n = 6–8 animals per group: sham-operated (sham), 2K1C surgery (2K1C), 2K1C surgery plus minipump implantation with Montelukast (577953-88-9 Merk, Darmstadt, Germany) (2K1C + ML), Oxgr1^−/−^ sham-operated (KO), or Oxgr1^−/−^ with 2K1C surgery (2K1C KO). 

### 4.2. 2K1C Goldblatt Surgery

The left renal artery was exposed via a left flank incision and isolated carefully. A silver clip with an internal diameter of 0.13 mm was placed around the left artery for 2 weeks to reduce whole-kidney blood flow and glomerular filtration in the clipped kidney (Figure 1A). The animals were allowed to recover for 4 weeks of the experimental period. A flank incision without clamping was used in control (sham) mice. After the surgery, the skin wound was cleaned with Betadine and closed with surgical clips. Mice were monitored in cages until they reached full recovery. 

### 4.3. Montelukast Administration

On day 0 (with or without 2K1C surgery), mice were lightly anesthetized with 2.5% isoflurane delivered in a box. Montelukast was administered at a dosage of 5 mg/Kg/day via osmotic minipumps for 14 days at a delivery rate of 0.5 μL/h (ALZET Model 2002, Cupertino, CA, USA) containing a vehicle of either 200 μL of saline or Montelukast. Montelukast was dissolved in 200 µL of saline. Osmotic minipumps were implanted under the back skin through a small skin incision. 

### 4.4. Colony of Oxgr1^−/−^

The Oxgr1 knockout mice with global deletion of the OXGR protein (KO in all figures and plots) were a gift from Dr. Joseph E. Kerschner (Medical College of Wisconsin). A colony of Oxgr1^−/−^ mice (C57BL/6J background) was established from breeding pairs of Oxgr1^+/−^ heterozygous mice. Male mice weighing 25–30 g (8 weeks of age) were used in all experiments. Heterozygous (Oxgr1^+/−^) mice and wild-type mice showed no difference in previous tests of blood pressure, water and food intake, and urinary flow, among others; thus, floxed and sham-operated mice were also used as controls. Genotype was verified by PCR reaction of tail genomic DNA. Primer pairs UTT069-21 (5′-GAGCCATGATTGAGCCACTG-3′) and UTT069-25 (5′-CACCACTGGCATAGTAATGG-3′) generated a 294 bp Oxgr1 specific fragment which presents in wild type but is absent in mutant allele, and UTT069-3 (5′-CAGAGCCATGCCTACGAG-3′) and GT (5′-CCCTAGGAATGCTCGTCAAGA-3′) amplified a 378 bp fragment specific to the selection cassette in the mutant allele. All animals were handled following the specific guidelines of the Institutional Animal Care and Use Committee at the Pontificia Universidad Católica de Valparaíso.

### 4.5. Blood Pressure Measurements

Systolic and diastolic arterial blood pressure was measured in all groups under a low dose of isofluorane allowing the animal to enter the chamber. The equipment (CODA system, Kent Scientific, Torrington, CT, USA) allowed for multifunctional monitoring capability (systolic, diastolic, and mean blood pressure as well as heart rate). The chamber was warmed at 36 °C for the process and mouse training was conducted 5 days. The measurements were taken at day 10 of treatment. 

### 4.6. Measurement of Urine Flow, Water Intake, Food Intake, and 24 h Sodium Excretion

C57BL/6 mice (12-week-old male) were placed in conditions of light–dark cycle (12 h), temperature of 21 °C, humidity of 50%, adequate ventilation, noise-free, food and water ad libitum during the protocols. On the day before the beginning of the protocol and on days 10 and 13, the animals were placed in metabolic cages (Rotarod, Ugo Basile, Washington, DC, USA) for 24 h for data collection of urine flow, water, and food intake. Animals were monitored every 3 to 6 h to check their health status during the whole protocol.

### 4.7. Saline Challenge Test

A saline challenge was performed on day 10 to evaluate the effect of 2K1C surgery on sodium balance and the effect of OXGR1 antagonism or its absence with or without 2K1C surgery. Mice were injected I.P. with a volume of warmed isotonic saline equivalent to 10% of their body weight and immediately placed in metabolic cages for urine collection. The results are expressed as the percentage of the injected sodium excreted over 5 h.

### 4.8. PRR Transcripts Quantitation by Real Time qRT-PCR

According to the manufacturer’s protocol, total mRNA was isolated from mouse renal tissues using RNeasy Mini Kit (Qiagen, Valencia, CA, USA). Total RNA was quantified using a nano-drop system. Quantitative real-time RT-PCR (qRT-PCR) was performed using the following primers: PRR: 5′-CAC AAG GGA TGT GTC GAA TG-3′, 3′-TTT GGA TGA ACT TGG GAA GC-5′, β-actin: 5′-ATC ATG AAG TGT GAC GTT GA-3′, and 3′-GAT CTT CAT GGT GCT AGG AGC-5′. Results are presented as the fold change ratio between the mRNA levels of the interest gene against the β-actin (“housekeeping” gene) compared to the control group.

### 4.9. Immunoblotting Analyses

A total of 40 μg of protein samples was electrophoretically separated on a precast NuPAGE 10% Bis-Tris gel (Novex) at 200 v for 45 min followed by semi-dry transference to a nitrocellulose membrane (Invitrogen) using iBlot (Invitrogen, Carsbad, CA, USA). Blots were blocked at RT for 3 h, incubated overnight with specific primary antibody at 4 °C, incubated with the corresponding secondary antibodies (1:5000 dilutions) at room temperature for 45 min, and then analyzed by normalization against β-actin, used as a housekeeping gene. PRR protein levels were detected using a polyclonal rabbit anti-PRR (ATP6AP2, 1:200; Cat. #HPA003156, Sigma-Aldrich, St. Louis, MO, USA) that recognizes the intracellular segment and the ectodomain. Tissue immunoblots are presented in each figure as representative images of n = 1 for each experimental group corresponding to n = 5–8. Results are presented as the ratio of PRR versus β-actin and presented as fold change of control.

### 4.10. Histology 

Sagittal sections of kidneys were fixed in Bouin (Sigma Aldrich, St. Louis, MO, USA) and embedded in paraffin. Then, 5 μm sections were prepared using a Leica RM2235 microtome (Leica Microsystems, Shanghai, China). The sections were washed and stained with hematoxylin–eosin (Sigma Aldrich, St. Louis, MO, USA). Images were captured with a Nikon digital sight DS-U3 digital camera, attached to a Nikon Upright Microscope ECLIPSE Ci-L (Nikon Instruments Inc., Melville, NY, USA), and analyzed using ImageJ 1.43u software (NIH, Bethesda, MD, USA).

### 4.11. Immunofluorescence of PRR and OXGR1 in Kidney Tissues 

Kidney slides of 3 μm were fixed and stained with anti-PRR at 1:200 dilutions (ATP6AP2, Cat. #HPA003156, Sigma-Aldrich, St. Louis, MO, USA), anti OXGR1 at 1:100 dilutions (Cat. # A11008, Invitrogen, Carlsbad, CA, USA), and detected with Alexa Fluor 488 or 594 conjugated to antirabbit IgG (Invitrogen, Life Science, Co.). The slides were mounted with ProLong^®^ Gold with 4,6-Diamidino-2-phenylindole dihydrochloride (DAPI) for nuclei staining. The images were obtained using a Nikon Eclipse-50i immunofluorescence microscope (Nikon Eclipse-50i, Tokyo, Japan) and were digitalized using the NIS-Elements BR version 4.0 from Nikon. Negative controls were obtained by omission of the specific primary antibody.

### 4.12. Measurements of α-Ketoglutarate

Tissue α-ketoglutarate was measured with Abcam α-ketoglutarate Assay Kit ab83431 (Abcam, Cambridge, UK) according to the manufacturer’s instructions. 

### 4.13. Ang II Medullary Kidney Content

Renal levels of Ang II were measured in kidney sample homogenates enriched in medullary tissues using ELISA Kit for Ang II (Cloud-Clone Corp, Katy, TX, USA) according to the manufacturer’s instructions.

### 4.14. ACE Enzymatic Activity

ACE catalytic activity was determined fluorometrically as described by Friedland and Silverstein and as modified by Ronchi et al. [58,59]. Freeze-dried samples were resuspended in 1 mL of 100 mM sodium borohydride buffer, pH 7.2, containing 340 mM sucrose and 300 mM NaCl, and a protease inhibitor cocktail (complete mini EDTA-free; Roche, Basel, Switzerland) was added. The protein concentration was determined using the Bio-Rad Protein Assay Dye Reagent Concentrate (Cat. no. #500-0006), according to the manufacturer’s instructions. ACE activity was determined by a fluorometric method using Z-Phe-His-Leu-OH as substrate. Briefly, 10 µL of the homogenate sample was incubated with the substrate diluted in borate buffer containing ZnSO_4_ at 37 °C, for 10 min. Enzymatic activity was interrupted by 0.28 M NaOH addition. The dipeptide His-Leu released by cleavage reacted with orthophthaldialdehyde, forming a fluorescent adduct. The reaction was stopped with 3 M HCl after 10 min. Fluorescence intensity was measured using a spectrofluorometer (Infinite^®^ 200 Pro, Tecan, Männedorf, Switzerland) with excitation at 360 nm and emission at 465 nm. ACE activity was corrected by protein concentration and expressed as nmol/min/mg of protein. 

### 4.15. Urinary Creatinine

Urinary creatinine was measured using Quidel Kit according to the manufacturer´s instruction (San Diego, CA, USA). Urine samples and standards were diluted to 1:40 and 25 µL was added to a 96-well plate along with 75 µL of colored solution. The reaction was stopped using 2 N H_2_SO_4_ and absorbance was read at 490 nm. 

### 4.16. Urinary Albumin

Urinary albumin was measured with the mouse albumin ELISA kit (Catalog # EEL119, Invitrogen, Carlsbad, CA, USA) according to the manufacturer´s instruction.

### 4.17. Plasma Renin Content

The produced angiotensin I (ng mL^−1^ h^−1^) was determined by ELISA (Angiotensin I Plasma Renin Activity ELISA, IBL International, Hamburg, Germany) and used to determine renin content in plasma from 4 ham and 4 mice subjected to the 2K1C surgery. Plasma measurements were performed after 2 weeks by collecting whole blood by trunk decapitation and following the manufacturer´s protocol. This protocol was used to corroborate the results in Figure 1E showing increased expression of renin in juxtaglomerular cells. 

### 4.18. Statistical Analysis

The results are expressed as mean ± SEM. Statistical analyses were performed using GraphPad Prism Software Version 8 (GraphPad Software, Inc., La Jolla, CA, USA). The normal distribution of each parameter analyzed was tested using Shapiro–Wilk. One-way ANOVA was used to compare the mean differences between groups. Post-test comparisons for two groups by non-paired (one-tailed) *t*-test were also used. A *p*-value < 0.05 was considered statistically significant.

## Figures and Tables

**Table 1:** Intrarenal angiotensin-converting enzyme (ACE) activity and Angiotensin II (Ang II) levels in right (non-clipped) and left (clipped, except in sham) kidneys from mice subjected or not to Goldblatt 2-kidneys 1-clip surgery for 2 weeks. * *p* < 0.05 versus sham group; ** *p* < 0.01 versus sham group.

	Sham WT	2K1C	2K1C + ML	2K1C KO	Sham WT + ML	Sham KO
ACE activity (nmol/min/mg)						
Right Kidney	6.8 ± 0.9	37.4 ± 11.2 **	34.4 ± 18.1 **	9.8 ± 0.8	6.3 ± 0.9	5.8 ± 1.2
Left Kidney	7.6 ± 1.1	17.2 ± 5.4 *	10.3 ± 3.2	7.1 ± 1.5	3.9 ± 1.1	4.6 ± 1.1
Ang II levels (ng/ml/total protein)						
Right Kidney	2.9 ± 0.3	23.4 ± 8.2 **	14.4 ± 18.1 *	4.1 ± 0.8	3.6 ± 0.6	3.7 ± 1.2
Left Kidney	2.5 ± 0.4	4.1 ± 0.4 *	3.3 ± 0.8	2.6 ± 0.9	2.1 ± 0.3	2.4 ± 1.1

## Data Availability

The authors will make all methods and materials, the experimental design to induce 2K1C Goldblatt hypertension, among others, available to other researchers, as requested.

## Supplementary Materials

The following supporting information can be downloaded at: https://www.mdpi.com/article/10.3390/ijms251810045/s1.
