# Supplementary figures and images for: OXGR1-Dependent (Pro)Renin Receptor Upregulation in Collecting Ducts of the Clipped Kidney Contributes to Na+ Balance in Goldblatt Hypertensive Mice
Source: Int J Mol Sci. 2024 Sep 18;25(18):10045. doi: 10.3390/ijms251810045 (PMC11432382; doi:10.3390/ijms251810045)

**Figure S1.** Right Kidney

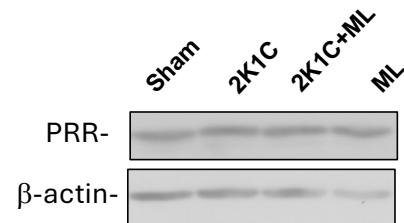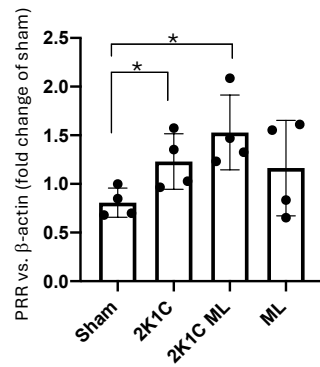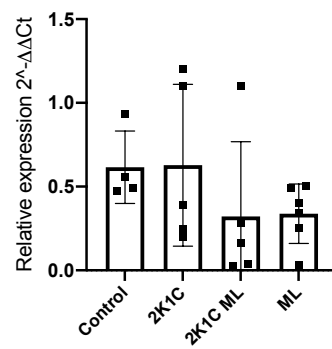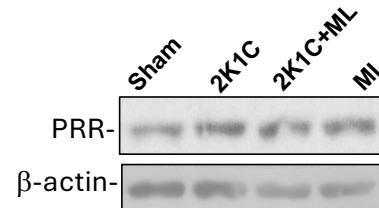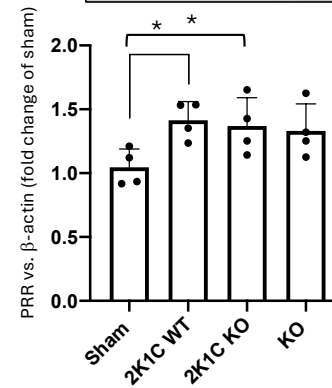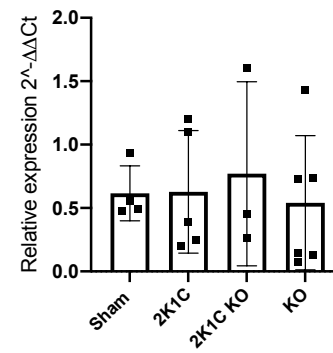

**Figure S2.** Right Kidney

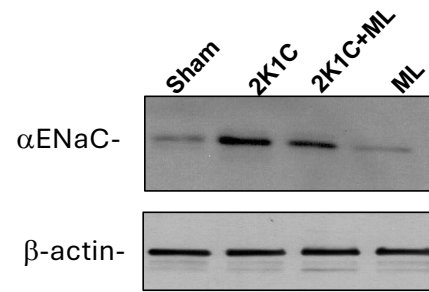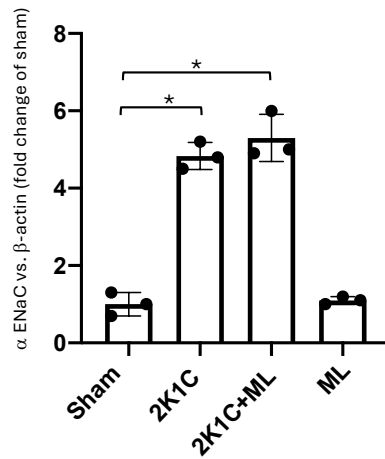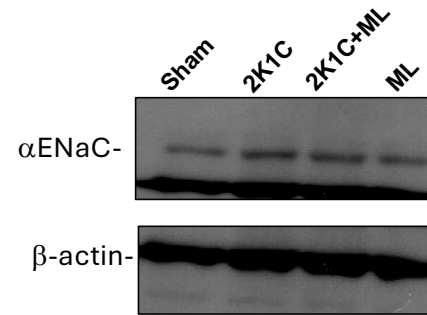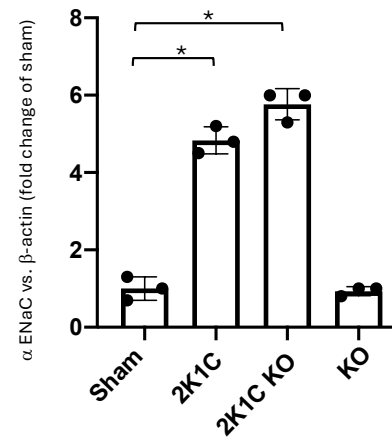

Supplement: Supplementary file 1 [file ijms-25-10045-s001.zip › ijms-3187076-supplementary.pdf]
